# The oral-gut axis: a missing piece in the IBD puzzle

**DOI:** 10.1186/s41232-023-00304-3

**Published:** 2023-11-06

**Authors:** Sho Kitamoto, Nobuhiko Kamada

**Affiliations:** 1 The World Premier International Research Center (WPI) Immunology Frontier Research Center (IFReC), 1012 IFReC Research Building, Osaka University, 3-1 Yamadaoka, Suita, Osaka 565-0871 Japan; 2 Division of Gastroenterology and Hepatology, Department of Internal Medicine, University of Michigan, 1150 West Medical Center Drive, Ann Arbor, MI 48109 USA; 3 Department of Pathology, University of Michigan, Ann Arbor, MI 48109 USA

**Keywords:** Systemic organ interactions, Periodontitis, Inflammatory bowel disease

## Abstract

Inflammatory bowel disease (IBD) is a multifactorial intractable intestinal disease. Focusing on only one facet of the pathogenesis of IBD is insufficient to fully capture the complexity of the disease, and results in limited advance in clinical management. Therefore, it is critical to dissect the interactions amongst the multifarious contributors to the pathogenesis to comprehensively understand its pathology and subsequently improve clinical outcomes. In this context, the systemic interactions between organs, particularly the oral-gut axis mediated by host immune cells and resident microorganisms, have garnered significant attention in IBD research. More specifically, periodontal disease such as periodontitis has been implicated in augmenting intestinal inflammation beyond the confines of the oral cavity. There is mounting evidence suggesting that potentially harmful oral resident bacteria, termed pathobionts, and pro-inflammatory immune cells from the oral mucosa can migrate to the gastrointestinal tract, thereby potentiating intestinal inflammation. This article aims to provide a holistic overview of the causal relationship between periodontal disease and intestinal inflammation. Furthermore, we will discuss potential determinants that facilitate the translocation of oral pathobionts into the gut, a key event underpinning the oral-gut axis. Unraveling the complex dynamics of microbiota and immunity in the oral-gut continuum will lead to a better understanding of the pathophysiology inherent in both oral and intestinal diseases and the development of prospective therapeutic strategies.

## Background

### Multifactorial pathogenesis of inflammatory bowel disease (IBD)

Inflammatory bowel disease (IBD) encompasses primarily two distinct subtypes of idiopathic intestinal disorders: Crohn’s disease (CD) and ulcerative colitis (UC). Both result in chronic inflammation within the gastrointestinal tract. IBD is typified by intermittent cycles of clinical relapse and remission [1–4]. Without proper management, chronic inflammation will cause irreversible damage to the intestines [5]. There is a growing body of evidence indicating that IBD emerges from an interplay between genetic predispositions, gut dysbiosis, and a variety of other factors including several environmental determinants [6]. However, none of these risk factors is sufficient to induce disease onset on its own. Therefore, further investigations are needed to elucidate the interplay of multiple known and as-yet-unidentified risk factors and gain a comprehensive understanding of the pathogenesis of IBD.

### The periodontal connection in the pathogenesis of IBD

**Fig. 1:**
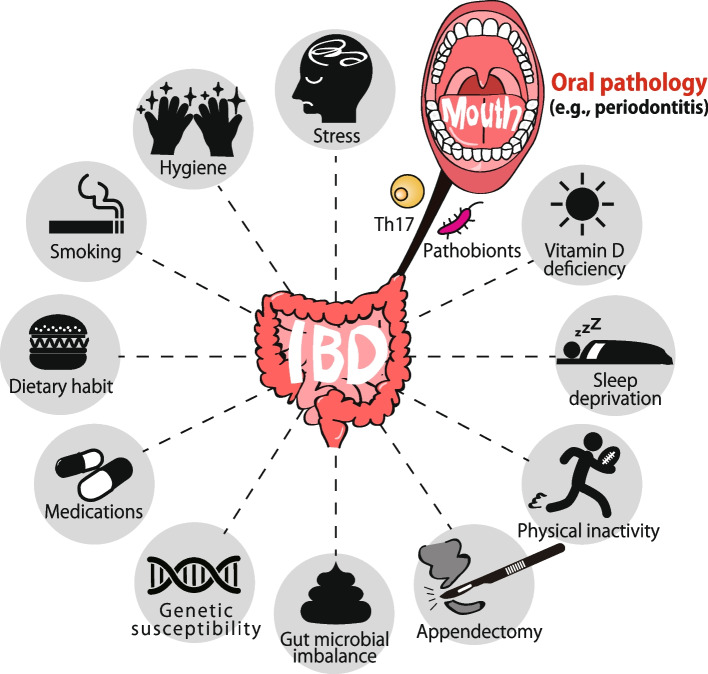
Oral pathology as a new risk for the development of inflammatory bowel diseases. Inflammatory bowel diseases (IBD) emerge from the confluence of multiple determinants including host genetic predisposition, environmental factors, immune dysregulation, and gut microbial configurations. Of these, oral pathology is newly identified as a potential risk factor for IBD development

The gut microbiota represents the most expansive bacterial community within the human body and is pivotal in maintaining host physiological homeostasis. This includes functions such as modulating the host immune system, facilitating nutrient digestion, and providing a barrier against colonization by pathogenic microorganisms [7–10]. Due to its critical role in orchestrating intestinal physiology, disruptions in the gut microbiota, commonly termed gut dysbiosis, have been implicated in the pathogenesis of IBD. Recent advancements in sequencing technologies have unearthed an anomalous enrichment of bacteria typically found in the oral cavity within the luminal contents and mucosal tissues of the intestines in IBD patients [11]. Given existing literature underscoring the detrimental impacts of certain oral resident bacteria (e.g., *Porphyromonas gingivalis* and *Fusobacterium nucleatum*) on intestinal homeostasis, there is a hypothesis that the oral cavity may act as a reservoir for oral pathobionts that can cause intestinal pathologies. This notion is supported by studies showing the similarity between oral and gut microbiota [12–14] and the increased prevalence of periodontitis in IBD patients when compared with healthy individuals [15, 16]. These observations suggest a potential correlation between periodontal disease and intestinal inflammation, possibly mediated by microbial translocation. However, it remains unknown whether the epidemiological relationship between oral and gut diseases is merely correlational or causal. In this vein, alongside recognized IBD risk factors [6], it has recently unveiled a causative link between periodontal inflammation and IBD pathogenesis (Fig. 1). This review aims to provide a synthesis of the prevailing knowledge on IBD pathogenesis, particularly emphasizing systemic organ interplay.

### Potential mechanisms of oral microbiota in the development of IBD

#### Bacteria-mediated pathway (Pathway 1)

Over the past two decades, there has been a significant surge in research investigating the impact of direct gut colonization by human-derived oral bacterial strains (e.g., *P. gingivalis*) on murine intestinal inflammation [1]. Nevertheless, due to inherent differences in the microbiota and host responses between humans and mice [17, 18], species mismatch models have some limitations. These limitations are especially prominent when delineating the precise causality of host–microbe interactions during the course of the disease within a single organism. Consequently, despite an extensive array of association-driven investigations, the causal nexus linking periodontal disease and intestinal pathologies remained elusive. To address this conundrum, a series of species-congruent experimental endeavors in mice has recently been made and unveiled the causal association between oral and intestinal regions during the development of intestinal inflammation [19]. Through an amalgamation of ligature-induced murine periodontitis and dextran sodium sulfate (DSS)-induced acute colitis models, the data discerned that mice with ligature-induced periodontitis developed a more pronounced colitis compared to their periodontitis-free counterparts. These observations further spotlighted the proliferation of oral pathobionts, especially *Enterobacteriaceae* (e.g., *Klebsiella spp.* and *Enterobacter spp.*), and their subsequent translocation to the gut being instrumental in intensifying colitis. Additionally, the propensity of these oral pathobionts to induce colitis was substantiated in the intestinal environment of genetically predisposed *Il10*–/– mice, albeit not in wild-type mice. Mechanistically, these findings showed that oral pathobionts elicit IL-1β production in the colonic mucosa by instigating the inflammasome in intestinal macrophages, thereby exacerbating intestinal pathology [19]. Leveraging the insights offered by a species-matched model, the authors delved deeper into deciphering the pivotal factors that govern efficient gut colonization by oral pathobionts. Remarkably, in a healthy gut, even the presence of periodontitis did not correspond to a discernible enrichment of oral pathobionts. However, in scenarios wherein both oral and gut inflammation coexisted, there was a notable upswing in gut colonization by these oral pathobionts. While further research endeavors are imperative to elucidate intricate mechanistic details, these insights, juxtaposed against prior studies emphasizing physiological barriers against ingested bacteria, allow us to advocate for a novel theoretical framework. We named it the “multiple-hit” hypothesis, elucidating the pathways facilitating the successful translocation of oral pathobionts to the gut during the progression of oral bacteria-mediated intestinal inflammation [19].

#### Host cell-mediated pathway (Pathway 2)

**Fig. 2:**
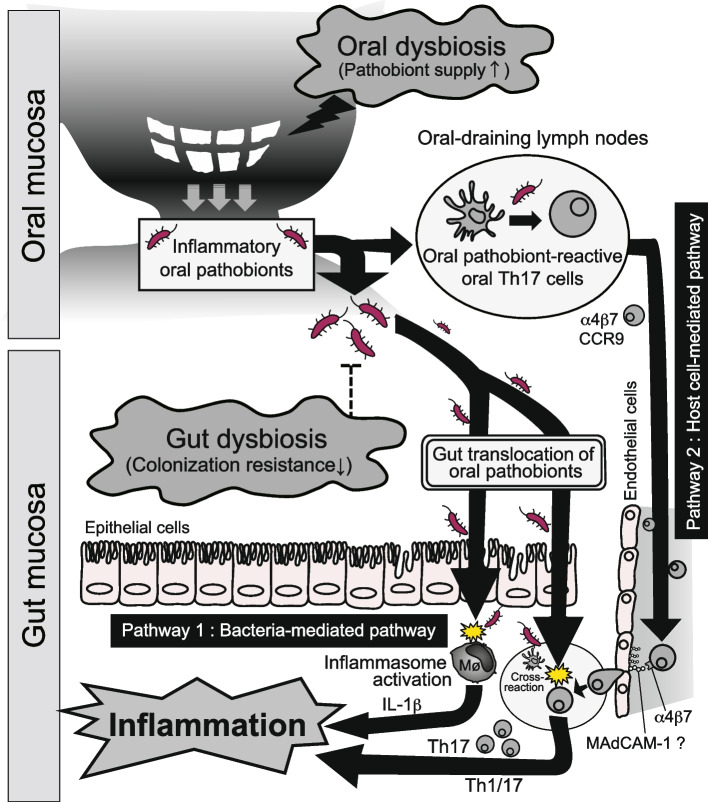
The proposed intricate nexus between the mouth and the gut during the gut inflammation: A microbial and immunological perspective. Bacteria-mediated pathway: certain oral pathobionts can be expanded in the inflamed oral cavity and reach the gut when colonization resistance given by gut commensals is perturbed. Upon entry of oral pathobionts into the intestine, they first penetrate the intestinal epithelium. Specific pathobionts can adhere to, and subsequently invade, epithelial cells. In the lamina propria, pathobionts can come across the immune cells such as macrophages, and activate inflammasome thereby fueling the gut inflammation via the release of proinflammatory cytokines (e.g., IL-1β). Host-cell mediated pathway: Concurrently with the direct migration of oral pathobionts to the gut as a result of co-existing oral and gut dysbiosis (as outlined in the above microbial pathway), the migration of oral immune cells to the gut is pivotal in the oral-gut axis during the pathogenesis of oral pathobiont-induced colitis. Mechanistically, during periodontal inflammation, Th17 cells primed orally can identify oral pathobionts (e.g., *K. aerogenes*) in the oral draining lymph nodes. These oral pathobiont-specific Th17 cells display gut-targeting molecules, including CCR9 and α4β7. Upon arrival in the gut, Th17 cells of oral origin can be stimulated by the translocated pathobionts, contributing to colitis. Given the phenotypic transition of oral Th17 cells to Th1 cells (evidenced in mice with periodontitis) and the simultaneous presence of the Th1-promoting factor IL-1β (produced by intestinal macrophages upon exposure to oral *K. aerogenes*, as shown in the above microbial pathway), it is plausible that both microbial and immunological pathways collectively exacerbate the intestinal pathology during oral pathobiont-driven gut inflammation

Existing literature robustly underscores that immune cells possess the capability to traverse from the gut to distant organs such as the liver, kidney, and joints, thereby playing a pivotal role in disease pathogenesis in these remote locales [20–22]. This trafficking of immune cells between the gut and other bodily regions appears to operate bidirectionally. Notably, reports indicate that leukocytes from the oral draining lymph nodes, especially the cervical lymph nodes (cLNs), can migrate to the gut even under steady-state conditions [23]. This phenomenon illuminates the overarching implications of systemic immune cell circulation in both health and disease. In line with this understanding, a recent study elucidates the pathological interplay of immune cells between the oral cavity and gut [19]. To elaborate, ligature-induced murine periodontitis augments susceptibility to acute DSS-induced colitis via direct gut colonization by oral pathobionts [19]. Intriguingly, despite the acute DSS-induced colitis model not allowing sufficient duration for the development of pathogenic T cells within the gut, Th17 and Th1 cells were significantly amplified within the colonic mucosa of ligature–DSS mice compared to DSS-only mice. Recognizing the well-documented cellular trafficking between the oral and gastrointestinal mucosae [23] and the instrumental role of Th17 in periodontal inflammation [24], it has been postulated that the pathogenic T cells amassed in ligature–DSS mice have their genesis in the oral cavity. To address this, the authors embarked on characterizing the immune reactions invoked by periodontitis within the oral milieu. Subsequent findings spotlighted the enrichment of CD3^+^CD4^+^CD44^hi^CD62L^lo^ effector memory T (T_EM_) cells in the cLNs of periodontitis mice. In harmony with prior observations [25], T_EM_ cells that accumulated in these mice displayed the IL-17A-producing RORγt^+^ Th17 phenotype. Deploying a co-culture system with oral antigen-loaded dendritic cells (DCs) and isolated orally primed Th17 cells, the authors identified that oral Th17 T_EM_ cells were responsive to oral pathobionts, notably *Klebsiella spp*. and *Enterobacter *spp., which proliferated exclusively in the inflamed oral mucosa. These revelations brought forth the pertinent query: Are oral Th17 cells capable of migrating to the gut mucosa? Comprehensive analysis revealed the expression of gut-homing markers, α4β7 integrin, and CCR9, on these cells, denoting their gut-directed propensity. Endeavoring for the evidence supporting the migration hypothesis, the authors employed in vivo photoconversion in transgenic mice expressing the Kaede protein [26]. Leveraging the inherent properties of the Kaede protein, which undergoes a color metamorphosis from green to red upon violet light exposure, consistent with prior reports [23], spotlighting Kaede-red CD4^+^ T cells in cLNs even in steady-state conditions. This offered incontrovertible evidence of orally primed Th17 cell migration to the gut mucosa. Importantly, the influx of oral Th17 cells to the gut witnessed a substantial uptick in DSS-treated mice. The precise intricacies governing this transmigration are yet to be fully decoded, but heightened expression of mucosal addressin cell adhesion molecule 1 (MAdCAM-1) in colonic lamina propria vessels, observed in IBD patients and specific animal models [27–29], insinuates the pivotal role of an augmented α4β7 integrin and MAdCAM-1 interplay in fast-tracking the oral Th17 cell influx into the inflamed gut (Fig. 2). Validating the colitogenic potential of oral Th17 cells within the gut, the authors unraveled that isolated oral Th17 cells instigated colitis upon intravenous introduction into *Rag1*−/− mice colonized by the oral pathobiont *K. aerogenes*. This was concomitant with an upsurge of Th17 (RORγt^+^) and Th1/Th17 (RORγt^+^ T-bet^+^) cells, contrasting with the non-colitis-inducing Kaede-green cells. Expanding on these insights, the study converges with existing literature emphasizing the paramountcy of Th17 cells in oral inflammation mediated by commensals [29–32]. In this context, while certain commensal-reactive Th17 cells generated in the gut appear to be non-pathogenic [33], specific circumstances in the gut can foster the emergence of IFN-γ-secreting Th1-like exTh17 cells that are capable of inducing profound intestinal inflammation [34, 35]. These findings underscore the trans-differentiation of oral pathobiont-reactive Th17 cells from a Th17 phenotype (RORγt^+^ T-bet^−^) to a Th1-like Th17 phenotype (RORγt^+^ T-bet^+^) upon entering the gut mucosa (Fig. 2). Given the clinical implications of Th1/Th17 cells, exploring this pathway behind oral-gut-associated colitis warrants dedicated attention in future research endeavors.

### Potential factors involved in the gut translocation of oral pathobionts during IBD

Given the critical role of ectopic gut colonization by oral pathobionts in both bacteria-mediated and host cell-mediated pathways (Fig. 2), gut translocation of oral pathobionts is conceivable to be a key event in the aforementioned oral-gut axis during IBD pathogenesis. This can be partitioned into three following critical interfaces: (1) The oral cavity—the source of oral pathobionts. (2) The gastrointestinal (GI) conduits—potentially facilitating the relocation of oral pathobionts from the mouth to the gut, and (3) The intestinal tract—where oral-derived pathobionts establish themselves.

#### The oral cavity as a reservoir of oral pathobionts

**Fig. 3:**
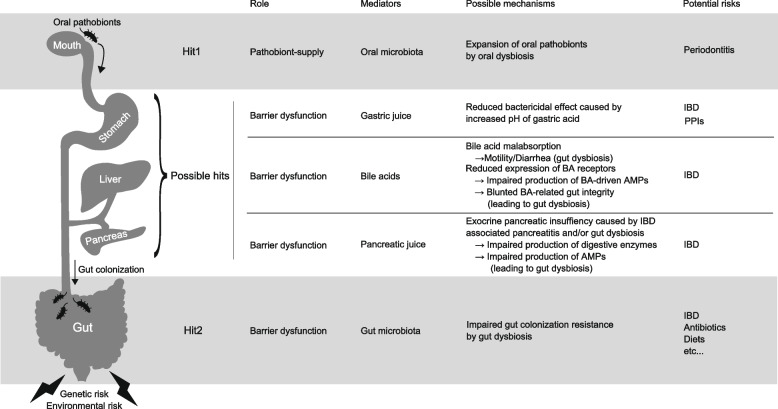
Multiple factors potentially involved in the gut translocation of oral pathobionts in IBD. The gut translocation of oral pathobionts is a central event modulating IBD pathology, dictated by the recently recognized oral-gut axis (Fig. 2). This can be partitioned into three critical interfaces: (1) The oral cavity—the source of oral pathobionts. (2) The gastrointestinal (GI) conduits—potentially facilitating the relocation of oral pathobionts from the mouth to the gut, and (3) The intestinal tract—where oral-derived pathobionts establish themselves. Each juncture presents promising avenues for therapeutic interventions in IBD by mitigating the gut translocation of oral pathobionts

Within the oral microenvironment, diseased oral conditions such as periodontitis lead to the formation of specific niches that influence microbial communities, thereby promoting the growth of particular oral pathobionts. With respect to this, recent research has discerned that oral dysbiosis, resulting from periodontal inflammation, becomes an initial step of oral-gut-associated gut inflammation by facilitating the significant expansion of colitogenic oral pathobionts in the oral cavity (Fig. 3). This, in turn, increases the chance of these orally derived pathobionts successfully transitioning to the intestine [19]. The data substantiates this, showcasing the simultaneous expansion of oral pathobionts, such as *Klebsiella* and *Enterobacter spp*., within both oral and gut compartments in mice subjected to ligature and DSS treatments. Conversely, there was no enrichment of these oral pathobionts in the intestine of mice without ligature, even when exposed to DSS treatment [36]. Furthermore, considering that nearly 25% of IBD incidences manifest during childhood or adolescence [37], factors inherent to oral health, beyond just periodontal inflammation, might also influence the proliferation of certain oral pathobionts. In a recent study, it was ascertained that the use of fixed orthodontic devices in school-aged children correlated with an elevated incidence of oral colonization by members of the *Enterobacteriaceae* family, encompassing *Klebsiella* and *Enterobacter *spp. [38]. While the clinical link between IBD and other oral conditions, such as orthodontics, remains to be elucidated, given the colitogenic potential of *Enterobacteriaceae* in both humans and mice [19, 39], this might suggest a heightened risk for IBD exacerbation. Intriguingly, the same study also revealed that children who habitually bit their nails and were undergoing orthodontic treatment exhibited the highest colony-forming units of these bacteria. This observation suggests that not only internal factors like inflammation or orthodontic treatments but also external behaviors like nail-biting can facilitate colonization by opportunistic environmental microorganisms, such as *Enterobacteriaceae*, within the oral cavity. This could, in turn, escalate the risk of gut inflammation attributable to pathobionts of oral origin.

#### The gastrointestinal conduits for facilitating the relocation of oral pathobionts from the mouth to the gut

The precise pathways by which oral pathobionts migrate to the gut remain to be fully elucidated. However, considering the anatomical continuity of the intestinal tract with the oral cavity, coupled with the constant exposure to both ingested foods and salivary secretions (approximately 1.5 L daily, containing approximately 1.5 × 10^12^ oral bacteria [40]), it stands to reason that oral pathobionts might transit to the gut via the enteral aspect of the gastrointestinal (GI) tract.

In this regard, compromised barrier functions within the GI continuum could influence the gut translocation of oral pathobionts. One salient factor is diminished gastric acidity, which prevents ingested bacteria from efficiently translocating to the distal GI tract [41, 42]. Previous research has evidenced that more than 99% of ingested microbes are inactivated as they pass through the stomach [43]. In support of this, increased gut colonization by oral bacteria, including genera such as *Streptococcus* and *Veillonella*, has been noted in patients manifesting gastric achlorhydria—a condition often induced by prolonged proton pump inhibitor (PPI) administration [44]. Similarly, patients diagnosed with gastroesophageal reflux disease (GERD) and undergoing sustained PPI treatment also demonstrate heightened oral bacterial populations in the gut, compared to healthy counterparts [39]. In terms of the potential influence of PPIs on IBD pathogenesis, prior studies have highlighted PPI usage as a possible risk determinant for unfavorable IBD outcomes [45, 46]. Notably, the gastric pH in IBD patients (with pH values for CD and UC listed respectively) is markedly elevated compared to the healthy population [47]. Moreover, given the reported pH range of 3–4 for the murine stomach [48], murine models might approximate the diminished gastric acidity seen in IBD patients more closely than that of healthy humans [19]. Nevertheless, these observations revealed negligible or diminished colonization by oral pathobionts in periodontitis-afflicted mice compared to mice presenting with both periodontitis and colitis, suggesting that merely reduced gastric acidity might not be the sole enabler for successful gut colonization by oral pathobionts [19]. Though the mechanism underlying the gastric pH is increased in IBD patients remains unknown and warrants further exploration, current data suggests a conducive role of diminished gastric acidity for the transit of ingested bacteria to the distal GI tract (Fig. 3).

Similarly, apart from their recognized immunomodulatory roles, bile acids, owing to their amphipathic detergent properties, disrupt bacterial cell walls, thus acting as effective antimicrobial barriers against pathogenic microbial invasions [49–51]. In line with this, previous research has identified alterations in bile acid profiles and gut microbial compositions in IBD patients. Specifically, concentrations of secondary bile acids, such as lithocholic acid (LCA) and deoxycholic acid (DCA), were markedly diminished, while levels of primary and conjugated bile acids surged in this patient cohort [52]. Intriguingly, while the exact mechanisms are yet to be deciphered, this elevated presence of primary and conjugated bile acids including cholic acid (CA), taurocholic acid (TCA), glycochenodeoxycholic acid (GCDCA), and taurochenodeoxycholic acid (TCDCA) correlated positively with an increased prevalence of certain oral pathobionts, notably *Klebsiella spp*. [19, 39, 53]. This suggests that bile acids could play a pivotal role in modulating gut colonization by oral pathobionts (Fig. 3).

Exocrine secretions from the pancreas, particularly the pancreatic juice, may also influence the gut colonization of oral pathobionts. Recent study has demonstrated that glycoprotein 2 (GP2), secreted by the pancreas into the intestinal lumen, thereby interacts with *E. coli* possessing FimH [54] which has been expressed on the surface of certain types of potentially pro-inflammatory commensal *E. coli* such as adherent-invasive *E. coli* [55–58]. In this study, the authors highlighted that a deficiency in pancreatic GP2 led to enhanced bacterial infiltration in the large intestine of colitis-afflicted mice when juxtaposed against their unaffected counterparts [54]. Moreover, the exogenous administration of recombinant GP2 (rGP2) effectively inhibited *E. coli* from breaching the intestinal epithelial barrier in colitic mice. These findings underscore the fundamental role of the pancreas-gut axis, wherein pancreatic GP2 functions as a primary defense mechanism against bacterial invasions into the intestines during episodes of gut inflammation. Although the association between pancreatic exocrine secretions and IBD pathogenesis has been suggested albeit the scarce and conflicting data [59], given the important role of colonization resistance (CR) conferred by commensal gut microbiota in averting the colonization by extra-intestinal microorganisms (as elucidated in the section of "Potential factors involved in the gut translocation of oral pathobionts during IBD" below) as well as the essential role of pancreatic enzymes in digesting the nutrients which fuel and shape the gut microbial configuration, the dysregulation in pancreas-gut axis might also play a role in regulating the gut translocation of oral pathobionts through the attenuation of CR in the gut (Fig. 3).

#### The gut microbiome as a last stand against the colonization of ingested extra-intestinal microorganisms

The CR provided by a balanced microbial composition within the healthy gut is of paramount importance in thwarting aberrant colonization by ingested oral bacteria [60, 61]. Recent reports have underscored the influence of multiple determinants, such as antibiotics, dietary patterns, and artificial sweeteners, in inducing gut dysbiosis, subsequently leading to a compromised gut CR. This perturbation enhances the propensity for oral bacteria to proliferate in gut-associated pathologies, including IBD [11, 61]. It can be posited, therefore, that the disturbance of gut CR that emerges as a consequence of gut dysbiosis serves as a subsequent mechanism facilitating the efficient gut colonization by oral pathobionts that have passed the gastric barriers (Fig. 3). Recent animal-based study corroborates this theory: mice with antecedent gut dysbiosis caused by DSS showed significant enrichment of oral pathobionts within the gut, only in the presence of periodontitis [19]. Aligning with these animal studies, a recent study utilizing matched saliva and fecal samples from IBD patients with and without periodontitis unveiled that the gut microbial profile in IBD patients bore a closer resemblance to their oral microbiota compared to the oral-gut microbial congruence observed in healthy subjects [62]. This lends weight to the premise that a pre-existing impairment in gut colonization is another critical event for the invasion of the gut by oral bacteria in IBD patients. It is noteworthy that the observations did hint at a potential deleterious interplay between periodontitis and intestinal inflammation in CD patients, although extensive validation encompassing a larger cohort and varied patient demographics is still needed [62]. In tandem with these findings, other research groups have reported the heightened colonization of specific oral microbes, such as *Streptococcus salivarius*, particularly in CD patients marked by a pronounced Crohn's Disease Activity Index (184.3 ± 2.9 vs. 67.1 ± 82.5, *P* = 0.012) and exacerbated disease manifestations (e.g., diarrhea, abdominal discomfort, hematochezia, fever, and fatigue, *P* = 0.016) [63]. Similar to the aforementioned animal-based insights, human study elucidated a pronounced enrichment of the *Enterobacteriaceae* family in CD patients with periodontitis compared to those without. However, within the confines of the study, the authors encountered limitations in pinpointing specific human oral pathobionts with an association to IBD. This was primarily attributed to a limited sample size and potential selection bias, given the relatively younger age bracket of participants, most of whom had manifested only incipient stages of periodontitis. Clearly, more rigorous study endeavors are warranted to discern the magnitude of the colitogenic potential of these accumulated bacterial populations in human subjects.

## Perspectives

In recent years, the exploration of oral microorganisms and their relationship with intestinal inflammation has markedly advanced. This progress is largely driven by studies emphasizing the implications of direct colonization by oral pathobionts in the GI tract [1]. The employment of murine models has also unveiled intricate intermucosal connections bridging the oral cavity and the gut [19]. Pathogenic T cells, once exposed to the oral environment, can migrate to the gut where they are reactivated by ingested oral pathobionts, aggravating intestinal inflammation. Despite these advancements, there remain significant knowledge gaps. For example, the pronounced microbial disparity between human and mouse models [64] raises concerns regarding the direct applicability of recent murine-based findings to human health. Pertinently, the colitogenic murine oral pathobionts, such as *K. aerogenes*, bear significant genetic resemblance to *K. aeromobilis*, a potent Th1-inducing colitogenic oral pathobiont isolated from the saliva of IBD patients [39]. While in-depth mechanisms have yet to be elucidated, the genetic and functional congruence of these species—particularly in the context of Th1-mediated immunity during gut inflammation—suggests that oral pathobiont-responsive immune cells may play a role in the pathogenesis of human IBD. Currently, there is no drug class or specific pharmaceutical agents targeting the oral-gut axis for the treatment of IBD. Enhanced understanding of the oral environment promises pivotal insights into the formulation of novel biomarkers and therapeutic interventions for intestinal inflammatory conditions. For instance, early detection of specific oral pathobionts could serve as an indicator for individuals predisposed to IBD onset or recurrence. Ensuring optimal oral hygiene to minimize oral pathobiont levels might mitigate disease progression in the gut and deter IBD development. Directing research towards the process of gut translocation by oral pathobionts presents another prospective intervention avenue. Limiting gut colonization opportunities by oral pathobionts could be realized by properly utilizing PPIs or antibiotics to maintain stomach and gut barriers against extraintestinal bacterial incursions. Indeed, PPI exposure has been linked to unfavorable clinical outcomes in patients with both UC and CD [45, 46]. Moreover, IBD patients on PPIs reportedly face challenges achieving remission when on infliximab [65]. Proper interventions for barrier dysfunctions, precipitated by bile acid malabsorption or exocrine pancreatic insufficiency, might help prevent IBD exacerbation rooted in the newly identified oral-gut pathology. Evidently, further research, especially focusing on the intricacies of inflammation instigated by oral pathobionts in the gut, will pave the way for innovative therapeutic approaches for IBD.

## Data Availability

Not applicable.
